# Integrated analysis of serum metabolomics and fecal microbiome in infants with necrotizing enterocolitis

**DOI:** 10.3389/fmicb.2025.1584041

**Published:** 2025-06-05

**Authors:** Zhi-ying Lin, Shan-shan He, Zi-tong Mo, Xiao-tian Liao, Zhou-shan Feng, Juan Kong, Lu Zhu, Ying Li, Hui-yuan Tan, Zhi-wen Su, Chun-hong Jia, Fan Wu

**Affiliations:** ^1^Guangzhou Key Laboratory of Neonatal Intestinal Diseases, Department of Neonatology, The Third Affiliated Hospital, Guangzhou Medical University, Guangzhou, China; ^2^Department of Obstetrics and Gynecology, The Third Affiliated Hospital, Guangzhou Medical University, Guangzhou, China; ^3^Guangdong Provincial Key Laboratory of Major Obstetric Diseases, Guangdong Provincial Clinical Research Center for Obstetrics and Gynecology, Guangzhou, China

**Keywords:** preterm infant, necrotizing enterocolitis, microbiome, metabolomics, multi-omics

## Abstract

**Background:**

Necrotizing enterocolitis (NEC), a lethal gastrointestinal disorder in preterm infants, remains poorly understood in its pathology, and early diagnosis are critically limited. Multi-omics approaches present unprecedented opportunities to elucidate NEC pathogenesis and identify clinically translatable biomarkers.

**Methods:**

Infants with Bell stage II-III NEC and gestational age-matched controls were enrolled. Serum/stool samples from NEC patients at acute (NEC-D) and recovery (NEC-R) phases, and controls (non-NEC) were collected. Fecal metagenomic sequencing and serum untargeted metabolomic profiling were performed. Clinical parameters were compared.

**Results:**

The study comprised seven NEC and seven non-NEC infants. Baseline neonatal characteristics and maternal perinatal parameters showed no significant differences between NEC-D and non-NEC except for markedly lower leukocyte counts in NEC infants. Fecal metagenomics revealed severely diminished alpha diversity in NEC-D versus both non-NEC controls and NEC-R, characterized with lower Chao1 index. NEC-D exhibited elevated *Escherichia coli* relative abundance alongside reduced *Staphylococcus haemolyticus*, *Staphylococcus aureus*, *Staphylococcus epidermidis*, and *Lactobacillus paracasei*. Correspondingly, KEGG functional gene analysis demonstrated impaired metabolism in NEC-D. Serum metabolomics identified significantly decreased ornithine, DL-arginine, L-threonine, leucine, and D-proline in NEC-D versus non-NEC. NEC-D also showed lower taurodeoxycholic acid, glycocholic acid, and chenodeoxycholic acid compared to NEC-R. Integrative analysis revealed a positive correlation between the metabolites D-proline and ornithine and the *Lactobacillus paracasei*, *Staphylococcus epidermidis*, and *Staphylococcus aureus* abundance.

**Conclusion:**

NEC is characterized by gut microbiota dysbiosis with reduced diversity, altered functional gene expression, and disrupted host-microbiota metabolic crosstalk. The identified serum metabolite-microbiome correlations provide mechanistic insights into NEC pathogenesis and potential diagnostic biomarkers.

## 1 Introduction

Necrotizing enterocolitis (NEC), a life-threatening gastrointestinal emergency in neonates, remains a leading cause of morbidity and mortality in neonatal intensive care units (Stoll et al., 2010). With an incidence rate of 5–10% among infants born before 32 weeks of gestation (Yee et al., 2012; Alsaied et al., 2020), this multifactorial disease disproportionately affects premature neonates and very low-birth-weight infants, carrying mortality rates exceeding 30% in severe cases (Jones and Hall, 2020; Hull et al., 2014). Current diagnostic protocols based on the revised Bell’s criteria (Agakidou et al., 2020) rely on radiographic evidence and clinical presentation, yet face significant limitations in differentiating early-stage NEC from common neonatal feeding intolerance. This diagnostic ambiguity underscores the urgent need for innovative strategies integrating advanced biological technologies to improve early detection and risk stratification.

Recent advances in multi-omics approaches—particularly the synergistic application of proteomics, metabolomics, microbiomics, and genomics—offer unprecedented opportunities to unravel NEC pathogenesis and identify clinically actionable biomarkers (Masi et al., 2024; Tian et al., 2024; Leiva et al., 2023). Among these, metabolomics has demonstrated significant potential in biomarker discovery across various clinical domains, including pediatric oncology, neurodevelopmental disorders, and perinatal medicine (Moco et al., 2013; Li et al., 2021; Ravi et al., 2022; Guo et al., 2022). However, NEC-specific metabolic research remains underdeveloped, with current studies limited to isolated biofluid analyses (blood, urine, or stool) that fail to capture systemic metabolic-microbial interactions (Morrow et al., 2013; Thomaidou et al., 2022; Thomaidou et al., 2019). Critical knowledge gaps persist regarding: (1) disease-specific metabolic signatures distinguishing NEC from other intestinal pathologies, and (2) the functional interplay between host metabolism and gut microbiota during NEC progression.

To address these limitations, this study implements a dual-omics framework combining non-targeted LC–MS/MS metabolomic profiling and metagenomic sequencing to characterize NEC-associated perturbations in both host metabolism and microbial ecology. By analyzing paired serum and fecal samples from NEC patients, we aim to: (1) identify clinically relevant metabolic biomarkers with diagnostic and prognostic value, and (2) elucidate microbiota-metabolite networks critical to NEC pathophysiology. This integrative approach advances beyond previous single-modality investigations, providing a systems-level perspective essential for developing precision diagnostic tools and targeted therapeutic interventions.

## 2 Materials and methods

### 2.1 Participant recruitment

This study enrolled neonates admitted to the Neonatal Intensive Care Unit at the Third Affiliated Hospital of Guangzhou Medical University between October 2022 and October 2023. The infants diagnosed with NEC stages II to III were eligible for inclusion in the NEC group. The control group consisted of non-NEC infants with a comparable gestational age at birth (±1 week), matched in a 1:1 ratio to the NEC group. Infants with the following conditions were excluded: significant congenital malformations, chromosomal anomalies, hereditary metabolic disorders, severe perinatal asphyxia, a family history of cow milk protein allergy or parental refusal to participate. This study was approved by the Clinical Research Ethics Committee of the Third Affiliated Hospital of Guangzhou Medical University (No. 2021-024). Written consent was diligently obtained from the parents of the infants included in this study.

### 2.2 Clinical data and biospecimen collection

The following demographic and clinical data were collected: (1) Maternal perinatal disorders and complications, including preeclampsia, premature rupture of membranes, and chorioamnionitis; (2) Neonatal demographic characteristics and birth conditions, including gender, gestational age, birth weight, mode of delivery, and birth asphyxia; (3) Treatments administered prior to NEC diagnosis, including antibiotic use, red blood cell transfusion, and mechanical ventilation; (4) Laboratory test results upon NEC diagnosis, including C-reactive protein (CRP) levels, white blood cell counts, and platelet counts.

Serum and fecal samples were collected from infants in the NEC group at two defined time points: (1) the acute phase (at diagnosis) and (2) the recovery phase (5–6 days after re-establishment of total enteral feeding). For the control group, samples were collected at a corresponding chronological age (±3 days) to matched NEC infants during their acute phase. Fecal samples were collected via anal or ostomy access, immediately stored at −40°C. Concurrently, 0.5 mL of venous blood was drawn, allowed to clot at room temperature for 30 min, and centrifuged at 2,500 rpm for 15 min. The resultant serum was aliquoted and stored at −40°C pending analysis.

### 2.3 Metagenomics

Metagenomic sequencing was used to analyze the fecal samples. The genomic DNA of the fecal microbiota was initially extracted using the CTAB (cetyltrimethylammonium bromide) method. Subsequently, the OD values of nucleic acids were assessed using a NanoDrop microspectrophotometer, followed by agarose gel electrophoresis. The dsDNA was fragmented into 50–1,000 bp fragments using the NEBNext DNA duplex fragmentation enzyme, with different action times. A fresh centrifuge tube, free of nucleic acids, was filled with the following: End Prep Enzyme Mix (3 μL), Buffer for End Repair Reaction (10X) (6.5 μL), fragmented DNA (55.5 μL), and the total volume was adjusted to 65 μL with the specified reagent. The components were thoroughly mixed using a pipette before initiating the reaction under the PCR cycle settings: 20°C for 30 s, 65°C for 30 s, and then held at 4°C.

For the subsequent steps, the following reagents were prepared and mixed thoroughly: Blunt/TA Ligase Master Mix (15 μL), NEBNext Adaptor for Illumina (2.5 μL), Ligation Enhancer (1 μL), and a total of 83.5 μL of reagent solutions. The mixture was incubated in a PCR instrument at 20°C for 15 min. Following this, 3 μL of USER enzyme was added to the mixture, thoroughly mixed, and allowed to incubate at 37°C for 15 min to facilitate adapter connection.

To further process the DNA fragments, AMPure XP beads were integrated into the system. After equilibrating the ligation product to room temperature, 13.5 μL dH2O was added to reach a total solution volume of 100 μL. Subsequently, 40 μL of AMPure XP beads were combined with the binding reaction solution and gently mixed using a pipette. The reaction tube was then left at room temperature for 5 min to transfer the supernatant to a new nucleic-acid free centrifuge tube. This process was repeated with additional AMPure XP beads for purification and drying of the resultant DNA fragments.

Further downstream, Adaptor Ligated DNA Fragments (23 μL), NEBNext High Fidelity 2X PCR Master Mix (25 μL), Index Primer (1 μL), Universal PCR Primer (1 μL), and a total volume of 50 μL were prepared into a reaction solution and thoroughly mixed. PCR reactions were executed under specific cycling conditions and purified using AMPure XP Beads. Finally, the library underwent quality checks, quantification using the ABI StepOnePlus Real-Time PCR System (Life Technologies), and subsequent sequencing based on pooling methods in line with the PE150 mode of Hiseq2500.

### 2.4 Non-targeted metabonomics

Serum samples were slowly thawed at 4°C, and an appropriate volume was added to a pre-cooled solution of methanol/acetonitrile/water (2,2,1, v/v) for vortex mixing. This was followed by a 30-min low-temperature ultrasound treatment, a 10-min incubation at −20°C, centrifugation at 4°C at 14,000 rpm for 20 min, vacuum drying of the supernatant, and reconstitution with 100 μL of an acetonitrile aqueous solution (acetonitrile: water = 1:1, v/v). The reconstituted samples were then subjected to mass spectrometry, vortexed, centrifuged at 4°C at 14,000 rpm for 15 min, and the supernatant was injected for analysis. The samples underwent separation on an Agilent 1290 Infinity LC Ultra Performance Liquid Chromatography System (UHPLC) HILIC column. Subsequently, the primary and secondary spectra of the samples were collected using an AB Triple TOF 6600 mass spectrometer.

The raw data were converted to MzML format using ProteoWizard, and then underwent peak alignment, retention time correction, and peak area extraction utilizing the XCMS program. Data extracted by XCMS were initially scrutinized for completeness, with metabolites showing over 50% missing values within any group being excluded from subsequent analyses. Null values were imputed using K-nearest neighbors (KNN) method, outliers were removed, and the data were ultimately normalized based on the total peak areas to ensure consistency across each sample and metabolite for parallel analysis.

### 2.5 Statistical analysis

Statistical analyses were performed using SPSS 26.0 software. Normally distributed continuous data were expressed as mean ± standard deviation (SD), with differences between two groups assessed using the independent samples t-test. Non-normally distributed continuous data were presented as median and interquartile range [M (IQR)], and differences between two groups were evaluated using the Mann–Whitney U test. Categorical data were displayed as counts and percentages [n (%)], with comparisons between two groups conducted using either the Pearson chi-square test or the Fisher exact test, depending on the sample size. A *p*-value of less than 0.05 was considered to indicate statistical significance.

## 3 Results

### 3.1 Clinical characteristics

Our study enrolled seven infants diagnosed with definite NEC (Bell’s stage II-III), two of whom required surgical intervention, along with seven gestational age-matched controls without NEC. Comparative analysis revealed no significant differences between NEC group and Control group in neonatal baseline characteristics, including gestational age at birth, birth weight, gender distribution, or Apgar scores at both 1- and 5-min assessments. Furthermore, maternal perinatal parameters showed comparable profiles between groups regarding delivery mode, antenatal glucocorticoid exposure, incidence of prolonged rupture of membranes ≥18 h, and pregnancy-related hypertensive disorders. Notably, the NEC group demonstrated significantly lower leukocyte counts compared to controls. No significant differences were observed in clinical management parameters including antibiotic utilization, mechanical ventilation duration, or laboratory markers such as C-reactive protein levels, platelet counts, and lactate concentrations, as shown in Table 1.

**Table 1:** Comparison of clinical characteristics between NEC group and control group.

	NEC group (*n* = 7)	Control group (*n* = 7)	*P*
Gestational age (weeks), M (Q1, Q3)	29.28 (27.51, 32.39)	30.28 (27.82, 32.5)	NS
Birth weight (grams), M (Q1, Q3)	1,130 (743, 1768)	1,472 (1,024, 1921)	NS
Postnatal age (days), M (Q1, Q3)	28 (11, 37)	24 (8, 36)	NS
Gender, *n* (%)			NS
Male	3 (42.9)	6 (85.7)	
Female	4 (57.1)	1 (14.3)	
Apgar score @ 1 min	7 (5, 9)	10 (9, 10)	NS
Apgar score @ 5 min	9 (7, 10)	10 (10, 10)	NS
Mode of delivery, *n* (%)			NS
Vaginal delivery	1 (14.3)	1 (14.3)	
Cesarean section	6 (85.7)	6 (85.7)	
Antenatal glucocorticoid, *n* (%)	7 (100)	5 (71.4)	NS
PPROM ≥ 18 h, *n* (%)	0 (0)	1 (14.3)	NS
Hypertensive disorder in pregnancy, *n* (%)	3 (42.9)	2 (28.6)	NS
Antibiotic use, *n* (%)	6 (85.7)	7 (100.0)	NS
Red blood cell transfusion, *n* (%)	2 (28.6)	3 (42.9)	NS
Duration of mechanical ventilation (days), M (Q1, Q3)	18 (5, 22)	23 (7, 29)	NS
White blood cell counts (10^9^/L), M (Q1, Q3)	6.32 (4.03, 9.24)	10.17 (9.26, 15.1)	0.047
Platelet counts (10^9^/L), M (Q1, Q3)	277 (262, 370)	380 (251, 481)	NS
C reactive protein (mg/L), M (Q1, Q3)	2.79 (0.50, 5.66)	0.7 (0.5, 1.03)	NS
Lactic acid (mmol/L), M (Q1, Q3)	1.05 (2, 2.35)	1.25 (2, 2.35)	NS

### 3.2 Fecal metagenomics analysis

Of the 21 initial fecal samples, 19 met inclusion criteria for further analysis. Two samples were excluded: one from the non-NEC group due to insufficient biomass and one from the acute NEC phase owing to excessive host DNA contamination (>50% host sequences). The final cohort comprised 6 acute-phase NEC cases, 7 convalescent-phase NEC cases, and 6 non-NEC controls.

#### 3.2.1 Alpha and beta diversity analysis

**Figure 1:**
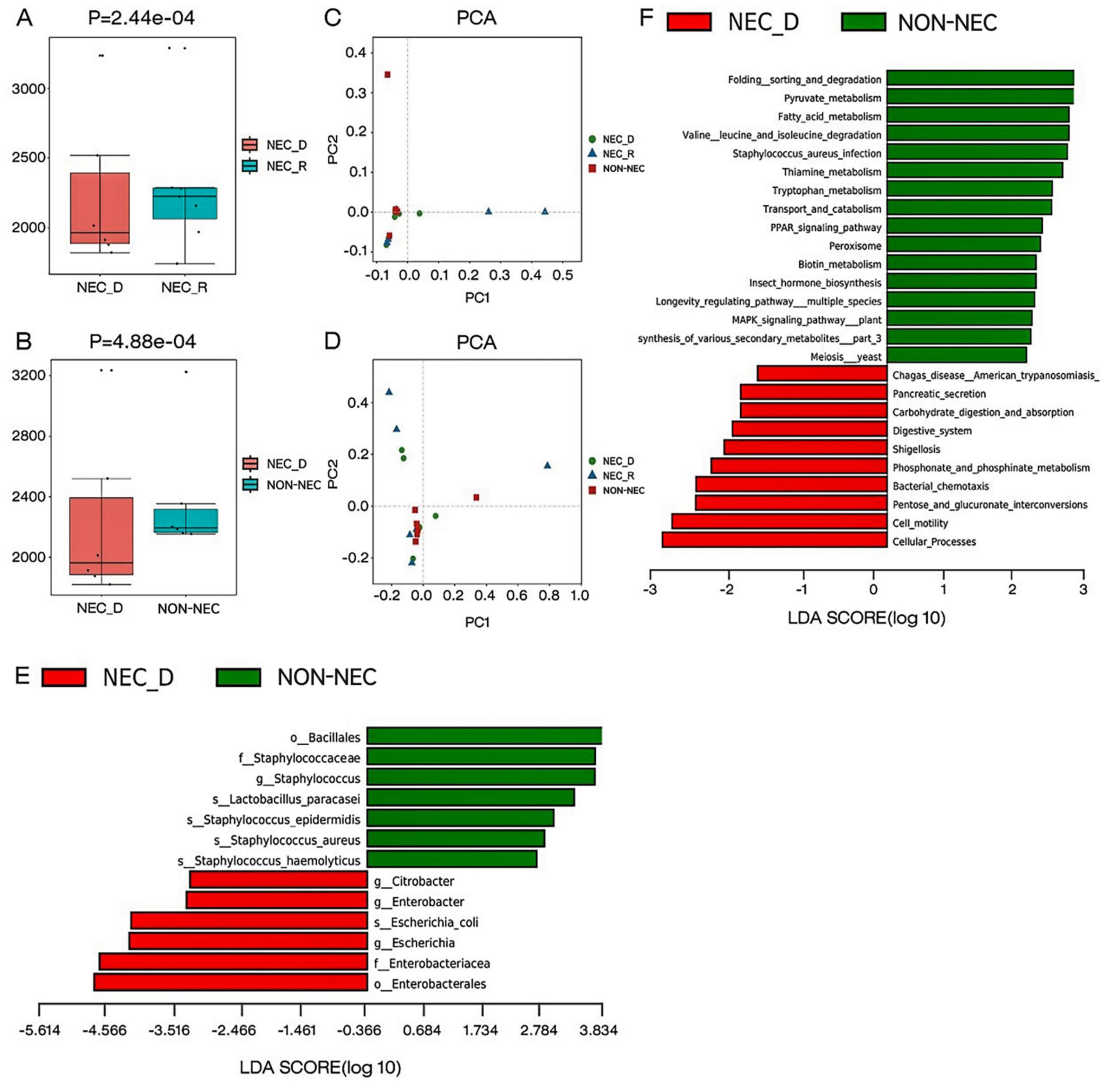
Different gut microbiota composition, diversity, functional genes between groups. **(A,B)** The alpha diversity as measured by the Chao1 index (the genus level). **(C)** Comparison of beta diversity between NEC-D, NEC-R, and non-NEC groups based on PCA analysis on genus level classification. **(D)** Comparison of beta diversity between NEC-D, NEC-R, and non-NEC groups based on PCA analysis on species level classification. **(E)** LEfSe analysis of specific bacteria in NEC-D and non-NEC groups. **(F)** Differential analysis of lefse KEGG function between NEC-D group and non-NEC group.

To detect differences in microbial community diversity, both alpha and beta diversity analyses were performed. Alpha diversity analysis revealed significant microbial community depletion during acute NEC episodes, as evidenced by markedly reduced Chao1 indices compared to both convalescent NEC (*p* < 0.05) and non-NEC groups (*p* < 0.05; Figures 1A,B). Beta diversity assessment through principal component analysis demonstrated no statistically significant separation in microbial composition across the three clinical states (Figures 1C,D).

#### 3.2.2 Abundance and composition of different bacteria between groups

To identify specific bacterial taxa associated with NEC, we performed a taxonomic comparison of gut microbiota between infants in the acute phase of NEC and healthy controls using linear discriminant analysis effect size (LEfSe). The analysis revealed 12 differentially abundant taxa (LDA score >2; Figure 1E), including two orders (Bacillales and Enterobacteriales), two families (Staphylococcaceae and Enterobacteriaceae), three genera (Citrobacter, Enterobacter, and Escherichia), and five species (*Staphylococcus haemolyticus*, *Staphylococcus aureus*, *Staphylococcus epidermidis*, *Lactobacillus paracasei*, and *Escherichia coli*).

#### 3.2.3 Functional gene differential analysis of bacterial flora

During comparative analysis of differential KEGG functions using LEfSe, significant functional disparities were identified. Infants in the acute phase of NEC exhibited reduced abundances of functional genes compared to non-NEC infants, particularly in pathways governing: folding, sorting, and degradation; pyruvate metabolism; fatty acid metabolism; valine, leucine, and isoleucine degradation; *Staphylococcus aureus* infection; thiamine metabolism; tryptophan metabolism; transport and catabolism; PPAR signaling; peroxisome activity; and biotin metabolism. Conversely, acute-phase NEC infants showed increased gene abundances related to carbohydrate digestion/absorption, phosphonate/phosphinate metabolism, pancreatic secretion, bacterial chemotaxis, cell motility, and pentose/glucuronate interconversions (Figure 1F).

### 3.3 Non-target metabolomics analysis

**Figure 2:**
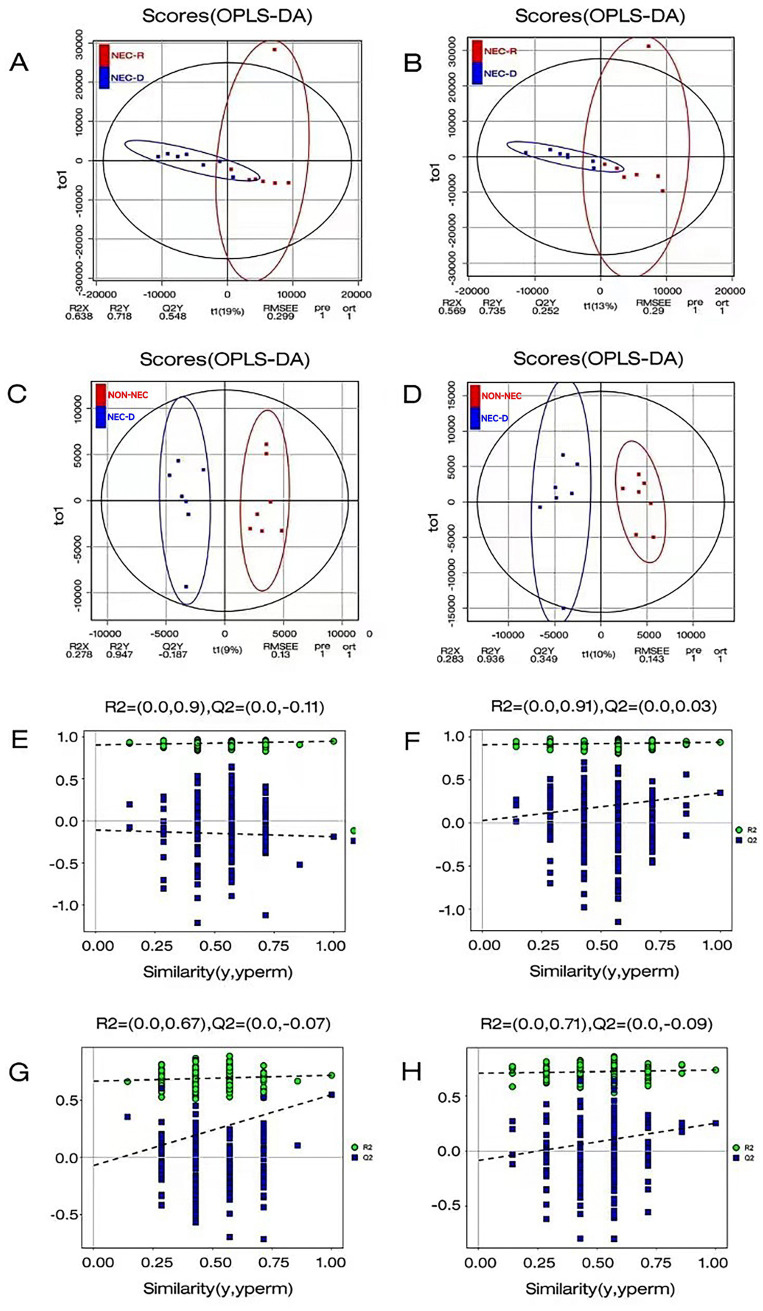
Orthogonal Partial Least Squares Discriminant Analysis (OPLSDA) was employed to perform a comprehensive statistical evaluation of the metabolite profiles within each group. **(A–C)** The OPLS-DA analysis was conducted in anionic mode between the two groups. **(B–D)** The OPLS-DA analysis was conducted in cationic model between the two groups. **(E)** OPLSDA permutation test comparison between NEC-D and non-NEC in anionic mode. **(F)** OPLSDA permutation test plot between NEC-D and non-NEC in cationic model. **(G)** OPLSDA permutation test plot between NEC-D and NEC-R in Anionic Mode. **(H)** OPLSDA permutation test plot between NEC-D and NEC-R in cationic model.

Serum samples were analyzed using non-targeted LC–MS/MS metabolomics. To gain a comprehensive understanding of metabolite expression and sample classification, orthogonal partial least squares discriminant analysis (OPLS-DA) was employed to develop a relationship model. OPLS-DA effectively discriminated among samples from different groups. Additionally, OPLS-DA permutation test plots were utilized to enhance intergroup information retrieval and to identify distinctive metabolites. Notably, in NEG, compared to the other three OPLS-DA models, the OPLS-DA model for the NEC-R and NEC-D groups demonstrated superior predictive capabilities and moderate predictive accuracy, as shown in Figure 2.

**Figure 3:**
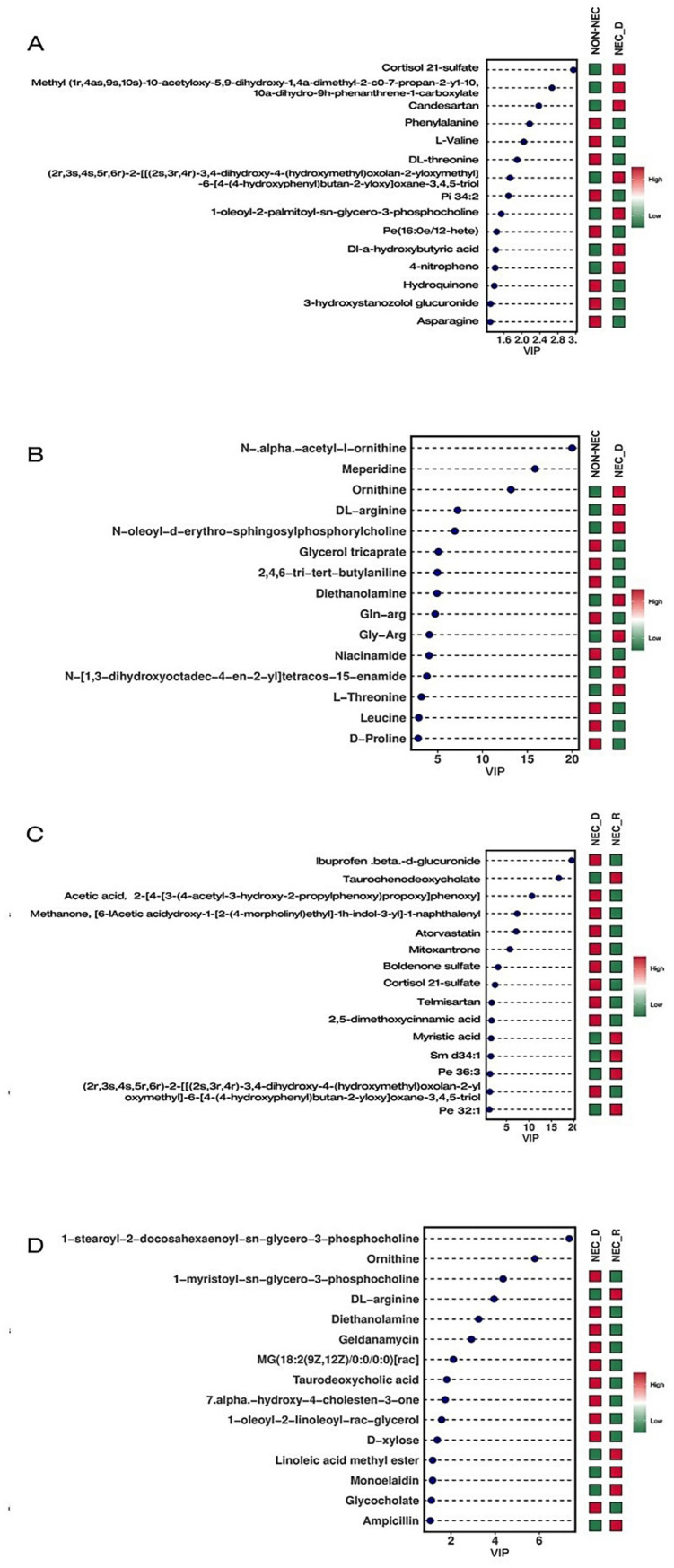
VIP diagram of differential metabolites. **(A)** Compare the differential metabolites in NEG between non-NEC and NEC-D. **(B)** Compare the differential metabolites in POS between non-NEC and NEC-D groups. **(C)** Compare the differential metabolites in NEG between NEC-R and NEC-D groups. **(D)** Compare the differential metabolites in NEG between NEC-R and NEC-D groups.

VIP (Variable Importance in Projection) values were calculated using the OPLS-DA model to identify the top 15 disparate metabolites in each group. Compared to non-NEC infants, NEC infants at the acute phase had lower levels of ornithine, DL-arginine, diethanolamine, nicotinamide, L-threonine, leucine, and D-proline; however, they had higher levels of candesartan and cortisol 21-sulfate. Meanwhile, compared to the recovery stage, infants at the acute stage of NEC had lower levels of ornithine, DL-arginine, diethanolamine, taurodeoxycholic acid, glycocholic acid, and chenodeoxycholic acid; but higher levels of atorvastatin, cortisol 21-sulfate, telmisartan, and 2,5-dimethoxycinnamic acid (Figure 3).

### 3.4 Combined metagenome and metabolome analysis

**Figure 4:**
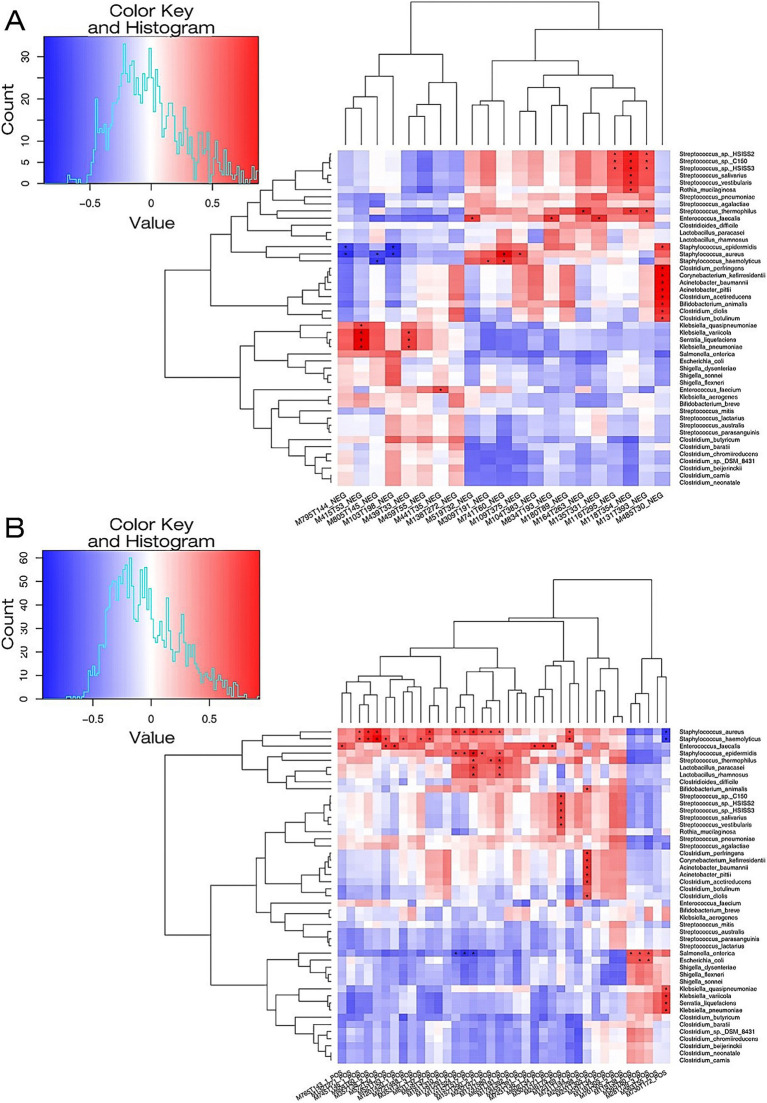
A heat map illustrating the correlation between microbial species and metabolites in the NEC-D group and the NON-NEC group. **(A)** The microbial species-metabolite correlation heat map in the context of NEG for both groups. **(B)** The microbial species-metabolite correlation heat map concerning POS among the groups. The metabolic names associated with the serial numbers of the metabolites depicted in the figure are fully detailed in Supplementary Table S1.

To enhance the understanding of the pathophysiological mechanisms of NEC, the relationship between differential metabolites in the serum and differential species in the fecal microbiome between the NEC group and the control group was further explored. Using R language, Pearson correlation analysis was conducted between differential metabolites and differential strain genes at the species level, with differential metabolites and strains screened using a significance level of *p* < 0.05. The findings revealed interesting correlations: N,N-dimethylaniline exhibited a positive correlation with *Escherichia coli*. Differential metabolites D-proline and ornithine showed strong positive correlations with *Lactobacillus rhamnosus*, *Lactobacillus paracasei*, Streptococcus epidermidis, and *Staphylococcus aureus*. Metabolites such as asparagine, DL-threonine, and L-valine were found to be positively associated with *Streptococcus* sp. C150 and Streptococcus hiss2 (Figure 4).

## 4 Discussion

Current diagnosis of NEC primarily relies on radiographic findings and clinical manifestations, yet still faces significant limitations in differentiating early-stage NEC. This diagnostic uncertainty highlights the critical need for novel approaches to improve clinical decision-making. Emerging multi-omics technologies now provide unprecedented potential to elucidate NEC pathogenesis and discover translational biomarkers for early detection. In this study, we employed integrated metagenomic and metabolomic analyses to characterize gut microbiota profiles and serum metabolite patterns across three cohorts: NEC patients at disease onset (NEC-D), non-NEC controls, and NEC patients at recovery (NEC-R). While no significant differences in microbial community composition were observed between groups, α-diversity indices revealed substantially reduced bacterial diversity in the NEC-D group compared to both non-NEC controls and NEC-R infants. Furthermore, NEC-D infants demonstrated decreased relative abundances of microbial functional genes involved in amino acid metabolism and oxidative stress response compared to non-NEC counterparts. Most notably, serum metabolomic profiling identified significant perturbations in the NEC-D group, including marked reductions in ornithine, DL-arginine, and diethanolamine concentrations relative to both control groups.

Growing evidence implicates gut microbiota dysbiosis as a key contributor to NEC pathogenesis. Longitudinal investigations have demonstrated temporal progression of microbial diversity in healthy preterm infants, while a developmental trajectory notably absent in NEC populations (Tarracchini et al., 2021; Dobbler et al., 2017). A 2023 meta-analysis consolidated these observations, confirming significantly diminished α-diversity in NEC patients compared to gestational age-matched controls (Pammi et al., 2017). Current consensus identifies characteristic microbial shifts in NEC development, particularly enrichment of Proteobacteria phylum members (including opportunistic pathogens such as *Klebsiella pneumoniae*, *Escherichia coli*, and Enterobacteriaceae) coupled with depletion of commensal Firmicutes and Bacteroidetes species (Pammi et al., 2017). Our findings align with these established patterns while providing novel insights. In our study, bacterial diversity was notably lower in the NEC-D group compared with non-NEC group or NEC-R group, which is supported by significant reduction in alpha diversity. Moreover, using Linear Discriminant Analysis Effect Size (LDA Effect Size, LefSe), our results identified the NEC group had lower relative abundance of *Staphylococcus haemolyticus*, *Staphylococcus aureus*, *Staphylococcus epidermidis*, and *Lactobacillus paracasei*, and higher relative abundance of *Escherichia coli* comparing with non-NEC group.

The pathobiont *Escherichia coli* has emerged as a critical microbial driver of NEC pathophysiology. Compelling evidence demonstrates that *E. coli* overgrowth disrupts gut ecological balance, reducing microbiota diversity and predisposing preterm infants to NEC (Zhang et al., 2025; Thänert et al., 2021). Notably, colonization by virulent strains such as uropathogenic *E. coli* (UPEC) significantly elevates NEC-associated morbidity and mortality (Ward Doyle et al., 2016). Recent longitudinal profiling revealed dynamic microbial shifts preceding NEC onset, with *E. coli* undergoing exponential expansion during the 24-h presymptomatic phase (Jenke et al., 2013) a pattern corroborated by our findings showing higher *E. coli* abundance in NEC-D versus controls. Mechanistically, *E. coli* may orchestrate NEC progression through dual ecological and metabolic perturbations. Metagenomic studies identify NEC-specific depletion of microbial enzymes governing tryptophan catabolism, biotin biosynthesis, and glycogenolysis pathways strongly correlated with *E. coli* dominance (Tarracchini et al., 2021). Our metabolomic data further implicate *E. coli*-derived N,N-dimethylaniline, a known immunomodulatory compound, in NEC-associated inflammation. These findings position *E. coli* as both a microbial biomarker and metabolic architect of NEC pathogenesis, with its enzymatic output potentially modulating host inflammatory cascades. Future multi-omics investigations should delineate strain-specific virulence factors and microbial-metabolite crosstalk underlying NEC progression.

Existing studies have demonstrated that single-omics approaches (e.g., metabolomics or microbiome analysis alone) are insufficient to capture the multidimensional pathophysiology underlying disease progression. Integrative multi-omics frameworks—synergizing proteomic, metabolomic, metagenomic, and genomic data—now enable systematic dissection of NEC mechanisms and identification of clinically translatable biomarkers. Our cross-domain correlation analysis, integrating fecal metagenomics with serum metabolomics, revealed three critical microbial-metabolite axes: (1) A strong positive correlation between *Escherichia coli* abundance and elevated N,N-dimethylaniline levels, potentially implicating microbial-derived aryl hydrocarbon receptor agonism in NEC pathogenesis (Deng et al., 2023; Lu et al., 2021); (2) Commensal *Lactobacillus rhamnosus* and *L. paracasei* demonstrated robust associations with D-proline and ornithine, metabolites functionally linked to enterocyte regeneration through nitric oxide synthase modulation (Mu et al., 2019; Gookin et al., 2002); (3) *Streptococcus* spp. abundance covaried with asparagine, DL-threonine, and L-valine concentrations, suggesting microbial regulation of immunomodulatory amino acid metabolism (Lavelle and Sokol, 2020; Morris et al., 2016; Sasabe et al., 2016). Future clinical translation should focus on validating these microbial-metabolite networks as therapeutic targets and developing rapid LC–MS/MS assays for bedside biomarker quantification.

However, limitations of this research include small sample sizes and fewer selected sample collection time points, which may impact the overall predictive performance of the OPLS-DA model. Moreover, the current experimental results lack validation from *in vivo* and *in vitro* experiments. Future endeavors should aim to minimize confounding variables between groups and enhance sample sizes. Both cell experiments and animal disease models are necessary to elucidate the causal relationship and analyze key functional genes between gut microbiota and host in NEC development. In summary, NEC is characterized by gut microbiota dysbiosis with reduced diversity, altered functional gene expression, and disrupted host-microbiota metabolic crosstalk. The identified serum metabolite-microbiome correlations provide mechanistic insights into NEC pathogenesis and potential diagnostic biomarkers.

## Data Availability

The data are publicly accessible in the NCBI repository under BioProject accession number PRJNA1258245: https://www.ncbi.nlm.nih.gov/bioproject/PRJNA1258245.
