# Supplementary material for: Integrated analysis of serum metabolomics and fecal microbiome in infants with necrotizing enterocolitis
Source: Front Microbiol. 2025 Jun 5;16:1584041. doi: 10.3389/fmicb.2025.1584041 (PMC12176818; doi:10.3389/fmicb.2025.1584041)
Supplement: Supplementary file 1 [file Table_1.docx]

***Supplementary Material***

**Table S1. The name corresponding to the metabolite in the heat map.**

| **serial number** | **The metabolic names** |
| --- | --- |
| M795T144NEG | Pe 40:4 |
| M415T53 NEG | Methyl (1r,4as,9s,10s)-10-acetyloxy-5,9-dihydroxy-1,4a-dimethyl-2-oxo-7-propan-2-yl-10,10a-dihydro-9h-phenanthrene-1-carboxylate |
| M805T145 NEG | 1-oleoyl-2-palmitoyl-sn-glycero-3-phosphocholine |
| M103T198 NEG | Dl-a-hydroxybutyric acid |
| M439T33_NEG | Candesartan |
| M459T55 NEG | (2r,3s,4s,5r,6r)-2-[[(2s,3r,4r)-3,4-dihydroxy-4-(hydroxymethyl)oxolan-2-yl]oxymethyl]-6-[4-(4-hydroxyphenyl)butan-2-yloxy]oxane-3,4,5-triol |
| M441T35 NEG | Cortisol 21-sulfate |
| M138T272 NEG | 4-nitrophenol |
| M519T32 NEG | 3-hydroxystanozolol glucuronide |
| M309T191 NEG | Prostaglandin f2.alpha. |
| M741T60 NEG | Pe(16:0e/12-hete) |
| M109T375 NEG | Hydroquinone |
| M104T383 NEG | DL-Serine |
| M834T193_NEG | Pi 34:2 |
| M180T89 NEG | Acamprosate |
| M164T263 NEG | Phenylalanine |
| M135T331 NEG | L-threonate |
| M116T295 NEG | L-Valine |
| M118T354_NEG | DL-threonine |
| M131T393 NEG | Asparagine |
| M485T30_NEG | Kendomycin |
| M765T143-1-POS | 2-docosahexaenoyl-1-palmitoyl-sn-glycero-3-phosphoethanolamine |
| M132T271-POS | Leucine |
| M745T146-1-POS | 1-stearoyl-2-linoleoyl-sn-glycero-3-phosphoethanolamine |
| M604T60-POS | 1-stearoyl-2-linoleoyl-sn-glycero-3-phospho-l-serine |
| M357T38-2-POS | Monoelaidin |
| M572T34-POS | Glycerol tricaprate |
| M521T93-POS | N-Palmitoylsphingosine |
| M120T420-1-POS | Threonine |
| M272T448-POS | Arg-Pro |
| M353T37-3-POS | Monolinolenin (9c,12c,15c) |
| M631T37-POS | N-[1,3-dihydroxyoctadec-4-en-2-yl]tetracos-15-enamide |
| M649T37-POS | N-tetracosenoyl-4-sphingenine |
| M161T310-POS | L-Threonine |
| M179T291-POS | 4,5-dihydroxy-3-propylcyclopent-2-en-1-one |
| M174T524-POS | Meperidine |
| M157T517-POS | D-Proline |
| M157T290-2-POS | N-.alpha.-acetyl-l-ornithine |
| M232T471-POS | Gly-Arg |
| M174T580-POS | Ornithine |
| M604T191-POS | 1,2-dioleoyl-sn-glycerol |
| M176T392-POS | Citrulline |
| M255T391-POS | His-Val |
| M743T146-1-POS | 1,2-dipalmitoyl-sn-glycero-3-phosphoethanolamine-n,n-dimethyl |
| M602T34-POS | 1-oleoyl-2-linoleoyl-rac-glycerol |
| M303T471-POS | Gln-arg |
| M246T75-POS | 2,4,6-tri-tert-butylaniline |
| M123T69-POS | Niacinamide |
| M741T44-POS | 1,2-dilinoleoyl-sn-glycero-3-phosphoethanolamine |
| M262T298-POS | (2r)-3-hydroxyisovaleroylcarnitine |
| M231T305-POS | DL-proline |
| M337T34-POS | MG(18:2(9Z,12Z)/0:0/0:0)[rac] |
| M116T305-POS | DL-arginine |
| M70T305-2-POS | Diethanolamine |
| M119T38-POS | Polygodial |
| M365T80-POS | 11.beta.,17.alpha.,20.beta.,21-tetrahydroxypregn-4-en-3-one |
| M281T39-3-POS | N,N-Dimethylaniline |
| M363T55-POS | Hydrocortisone |
| M730T172-POS | N-oleoyl-d-erythro-sphingosylphosphorylcholine |
